# Identification of chicken CAR homology as a cellular receptor for the emerging highly pathogenic fowl adenovirus 4 via unique binding mechanism

**DOI:** 10.1080/22221751.2020.1736954

**Published:** 2020-03-16

**Authors:** Qing Pan, Jing Wang, Yulong Gao, Qi Wang, Hongyu Cui, Changjun Liu, Xiaole Qi, Yanping Zhang, Yongqiang Wang, Kai Li, Li Gao, Aijing Liu, Xiaomei Wang

**Affiliations:** a Division of Avian Infectious Diseases, State Key Laboratory of Veterinary Biotechnology, Harbin Veterinary Research Institute, Chinese Academy of Agricultural Sciences, Harbin, People’s Republic of China; b Jiangsu Co-innovation Center for the Prevention and Control of Important Animal Infectious Diseases and Zoonoses, Yangzhou University, Yangzhou, People’s Republic of China

**Keywords:** Emerging FAdV-4, novel binding mechanism, cell receptor, chicken CAR, D2 domain, short fibre

## Abstract

Since 2015, the prevalence of severe hepatitis-hydropericardium syndrome, which is caused by the novel genotype fowl adenovirus serotype 4 (FAdV-4), has increased in China and led to considerable economic losses. The replication cycle of FAdV-4, especially the emerging highly pathogenic novel genotype FAdV-4, remains largely unknown. The adenovirus fibre interacts with the cellular receptor as the initial step in adenovirus (AdV) infection. In our previous studies, the complete genome sequence showed that the fibre patterns of FAdV-4 were distinct from all other AdVs. Here, protein-blockage and antibody-neutralization assays were performed to confirm that the novel FAdV-4 short fibre was critical for binding to susceptible leghorn male hepatocellular (LMH) cells. Subsequently, fibre 1 was used as bait to investigate the receptor on LMH cells via mass spectrometry. The chicken coxsackie and adenovirus receptor (CAR) protein was confirmed as the novel FAdV-4 receptor in competition assays. We further identified the D2 domain of CAR (D2-CAR) as the active domain responsible for binding to the short fibre of the novel FAdV-4. Taken together, these findings demonstrate for the first time that the chicken CAR homolog is a cellular receptor for the novel FAdV-4, which facilitates viral entry by interacting with the viral short fibre through the D2 domain. Collectively, these findings provide an in-depth understanding of the mechanisms of the emerging novel genotype FAdV-4 invasion and pathogenesis.

## Introduction

Adenoviruses (AdVs) belong to the *Adenoviridae* family and are non-enveloped, double-stranded DNA viruses [1]. The International Committee on Taxonomy of Viruses [2] separates *Adenoviridae* into five genera: *Mastadenovirus*, *Aviadenovirus*, *Siadenovirus*, *Atadenovirus*, and *Ichtadenovirus*. Human adenoviruses (HAdVs) are common human pathogens that cause diseases affecting mainly the eyes, airways, and gastrointestinal tract. Six types of HAdVs (HAdV-8, -19, -37, -53, -54, and -56) cause severe epidemic keratoconjunctivitis [3–5], which affects 20–40 million individuals worldwide every year. Fowl adenoviruses (FAdVs) are associated with several notable diseases in chickens and other birds, such as inclusion body hepatitis [6], hepatitis-hydropericardium syndrome (HHS) [7], and gizzard erosion [8]. These diseases cause significant economic losses in the poultry industry across the world. Notably, severe HHS, which is caused by a highly pathogenic novel FAdV genotype (FAdV-4) emerged in China in June 2015 [9,10]. Although different serotypes of pathogenic FAdVs have been found to induce severe diseases, the pathogenesis of FAdVs, especially the emerging FAdV-4 genotype, is relatively unknown.

Interactions between viruses and host cell surface receptors represent the initial step in viral infection, which directly or indirectly affects pathogenesis. As reported, AdV capsid fibre/fibres proteins bind to the cell surface receptor and initiate infection [11,12]. Most AdVs have one unique fibre that attaches to variable cellular receptors. HAdV-2 and HAdV-5 in subgroup C use the high-affinity human coxsackie and adenovirus receptor (CAR) as the cellular receptor to infect host cells [13,14], HAdV-37 uses integrins to infect human corneal cells [15], mouse adenovirus type 1 utilizes integrin and heparin sulfate as cellular receptors [16], bovine adenovirus serotype 3 utilizes sialic acid as a receptor for viral entry [17], and porcine adenovirus serotype 3 internalization is independent of CAR and integrin, but uses some other receptors [18]. However, HAdV-52, HAdV-40 [19], and HAdV-41 [20] are equipped with two different fibre proteins, only the long fibre type is inserted into each penton and binds the CAR, whereas the short fibre binds to sialylated glycoproteins during infection [21,22].

FAdVs are members of the *Aviadenovirus* genus and are separated into either five species (designated FAdV-A to FAdV-E) or twelve serotypes (designated FAdV-1 to FAdV-8a and FAdV-8b to FAdV-11) based on molecular criteria and serum cross-neutralization tests [23]. The fibre patterns of the twelve FAdV serotypes show high diversity. FAdV-B (serotype FAdV-5), FAdV-D (serotypes FAdV-2, -3, -9, and -11), and FAdV-E (serotypes FAdV-6, -7, -8a, and -8b) each have a unique single fibre, whereas FAdV-A (serotype FAdV-1) and FAdV-C (serotypes FAdV-4 and -10) have two distinct fibres inserted in each penton base. Although both the emerging novel genotype FAdV-4 and FAdV-1 have two fibres in each penton, their fibre patterns are differ significantly [24]. Long fibre 1 of CELO (an FAdV-1 type strain) has 710 amino acids (aa), whereas short fibre 2 has 410 aa [25]. In contrast, fibre 1 of the novel FAdV-4 has 431 aa, whereas fibre 2 has 479 aa [24], suggesting that fibre 1 might be the short fibre of FAdV-4. The unique fibre pattern of FAdV-4 implies that a novel fibre–receptor-interaction mechanism occurs in FAdV-4-infected host cells. Given that the cellular receptor of the pathogenic novel FAdV-4 is unknown, gaining insight into the molecular details underlying interactions between FAdV-4 and host cells is essential for obtaining a deeper understanding of its pathogenesis.

In this study, the chicken CAR homolog was identified as a cellular receptor for the emerging novel FAdV-4, via a unique binding mechanism. The novel FAdV-4 utilized its short fibre to bind D2-CAR, in contrast to previous studies showing that adenovirus uses its unique or long fibre to bind D1-CAR. Herein, we reveal a novel binding mechanism associated with FAdV-4 infection and provide important information for an in-depth understanding of the invasion and pathogenic mechanisms of the novel FAdV-4.

## Materials and methods

### Cell culture, viruses, and antibodies

Chicken LMH cells were cultured in Dulbecco’s modified Eagle’s medium (DMEM) (Sigma-Aldrich) supplemented with 10% fetal bovine serum (FBS) (Gibco), 100 IU/ml penicillin, and 100 μg/ml streptomycin. HEK293 and PK-15 cells were grown at 37°C in DMEM (Sigma-Aldrich) supplemented with 10% FBS in an atmosphere containing 5% CO_2_. The FAdV-4 strain, HLJFAd15, was identified and preserved in our laboratory (GenBank^TM^ KU991797) [26]. Anti-HA tag and anti-human Fc antibodies were purchased from Sigma-Aldrich. Custom mouse pAbs against the FAdV-4 fibre 1, FAdV-4fiber 2, and chicken CAR proteins were obtained from Biodragon Immunotechnologies Company. A mouse anti-Hexon Mab was derived from GenScript Company. IRDye 800 CW donkey anti-mouse IgG (H + L) was purchased from Li-COR Biotechnology.

### Expression and purification of recombinant proteins

The recombinant fibre 1 protein was expressed in *Escherichia coli*. A gene fragment encoding the full-length fibre 1 protein was cloned into the prokaryotic expression vector, pET48a. The recombinant plasmid was transformed into BL21 (DE3) competent cells (TransGene) for inducible expression. The proteins were purified by Ni Sepharose Excel resin (GE Healthcare), following the manufacturer’s recommended protocol. The fibre 2 protein, the CAR protein, and the ECD, D1, and D2 domains of CAR were transiently expressed by transfecting Expi293F cells (Thermo Fisher Scientific) using Polyfectine (Sigma-Aldrich) [27]. Cell culture supernatants were harvested at 5 days post-transfection and centrifuged at 10,000 × *g* for 30 min to remove cell debris. The supernatants were sterile-filtered and purified with a protein A resin (GenScript Company). The expression of recombinant proteins was detected by SDS-PAGE and western blot analysis, using appropriate antibodies.

### DNA and RNA extraction, and qPCR

Viral DNA was extracted from cells using the AxyPrep Body Fluid Viral DNA/RNA Miniprep Kit (Qiagen) according to the manufacturer’s instructions. RNA was extracted from cells using the RNeasy Mini Kit (Qiagen) per the manufacturer’s instructions. One microgram of RNA was reverse transcribed to complementary DNA (cDNA) using the ReverTra Ace qPCR RT Master Mix with gDNA Remover (Toyobo) in a 20-μl reaction mixture. The sequences of the primers and probes used for qPCR analysis of FAdV-4 and chicken CAR are shown in Table 1.

**Table 1.** Primer and probe sequences used for qPCR analysis of the novel FAdV-4 and chicken CAR homolog.

Target	Probe and primer sequences (5′–3′)
FAdV-4	Probe: 5′-FAM-TCTGTCGTGACATTTCGGGTGGG-3′-TAMRA
	Primer F: 5′-CAGTTCATTTCCGCCACC-3′
	Primer R: 5′-GCAGCCGTTGAGCCTTTT-3′
CAR	Probe: 5′-FAM-TGCTTCAAACCGAGTTGGCACAGA-3′-TAMRA
	Primer F: 5′-TGCCACTTCCGTACTAAACAAA-3′
	Primer R: 5′-AGAATAGCTCCAGCAATTACACC-3′

DNA and cDNA samples were analyzed by qPCR, which was performed using Premix Ex Taq (Probe qPCR) (Takara) under the following conditions: initial denaturation at 95°C for 5 min, followed by 45 cycles of denaturation at 95°C for 10 s and elongation at 65°C for 40 s. The fluorescent signal was collected during the elongation step. All standards, controls, and infected samples were examined in triplicate on the same plate. The cDNA copies were normalized to the number of 28S cDNA copies measured in the same samples. The 2^−ΔΔCt^ method was used for data analysis for the relative quantification of CAR. The FAdV-4 virus load was measured quantitatively by the absolute-analysis method.

### Blocking assay

To neutralize the FAdV-4 infection, the virus (MOI = 0.1) was pre-incubated for 1 h at 4°C with different dilutions of recombinant proteins (CAR, D1, D2, and ECD), a pAb against fibre 1 or fibre 2, or the related elution buffer as a negative control. The mixture was added to LMH cells for 1 h at 37°C. The cells were washed 3 times with phosphate-buffered saline (PBS; pH 7.0) and then cultured at 37°C. The samples were collected at 24 hpi and the virus copy numbers were detected by qPCR [28].

In the blockade assay, LMH cells were incubated with different dilutions of the CAR pAb (or mouse IgG as a negative control), or the recombinant fibre 1 or fibre 2 protein for 1 h at 37°C. This was followed by two washes with PBS (pH 7.0). Thereafter, the cells were inoculated with FAdV-4 (MOI = 0.1) for 1 h at 37°C. The cells were washed 3 times with PBS (pH 7.0) and then maintained at 37°C for another 24 h. The samples were collected at 24 hpi to detect the copies of FAdV-4.

### Protein precipitation and MS analysis

LMH cells were washed with PBS (pH 7.0) and lysed with Western and IP lysis buffer (Beyotime Biotechnology) at 4°C for 30 min. Cell lysates were incubated for 10 h with Fc-tagged fibre 1 or Fc protein (as a negative control) and protein A/G agarose antibody. Precipitates were washed five times in Western and IP lysis buffer and once in PBS, and subsequently centrifuged 5000 rpm for 5 min (Eppendorf). The proteins binding in agarose were separated by SDS-PAGE and visualized by silver staining (Thermo Fisher Scientific) according to the manufacturer’s instructions. Differential bands between lysate incubated with fibre 1 and negative control lysate were excised from the gel and transferred to Shanghai Hoogen Biotechnology Company for mass spectrometry (MS) analysis.

### Confocal imaging

LMH cells were plated on cell-imaging dishes (Eppendorf) and transfected with the pCAGGS-HA-CAR vector. After 24 h, the cells were adsorbed with FAdV-4 (MOI = 1) and allowed to bind at 4°C for 1 h. The cells were then washed 4 times with PBS. In another experiment, 293 T cells were plated on cell-imaging dishes (Eppendorf) and transfected with the pCAGGS-HA-CAR vector. After 24 h, the cells were infected with FAdV-4 (MOI = 1) and maintained at 37°C for 24 h.

The cells were fixed with 4% formaldehyde for 30 min and then washed with PBS. Both types of cells (LMH and 293 T) were treated with 1% Triton X-100 for 5 min and then washed with PBS. Thereafter, the cells were incubated for 1 h with an FAdV-4 Hexon Mab (1:200) and an HA-tag antibody in PBS, and then washed three times with PBS. The samples were stained with DAPI to visualize the cell nuclei, with Goat anti-Mouse IgG (H + L) Cross-Adsorbed Secondary Antibody Alexa Fluor 488 (Invitrogen) (1:200) to visualize FAdV-4, and Goat anti-Rabbit IgG (H + L) Cross-Adsorbed Secondary Antibody, Alexa Fluor 546 (Invitrogen) (1:200) to visualize HA-tagged CAR. The cells were incubated for 1 h with secondary antibodies in PBS and then washed 4 times with PBS. The samples were imaged on an LSM880 (Zeiss) Confocal Laser Scanning Microscope with Fast Airyscan using ZEN 2 software.

### RNAi and RNA-overexpression assays

The following siRNAs were used to study CAR downregulation: negative control, sense 5′-UUCUCCGAACGUGUCACGUTT-3′, antisense 5′-ACGUGACACGUUCGGAGAATT-3′; CAR-gga-651: sense 5′-CCUGCCACUUCCGUACUAATT-3′, antisense 5′-UUAGUACGGAAGUGGCAGGTT-3′; CAR-gga-973: sense 5′-GCAGCUACAUAGGCAGCAATT-3′, antisense 5′-UUGCUGCCUAUGUAGCUGCTT-3′; CAR-gga-1047: sense 5′-CCAUAUAGCCAGGUUCCAATT-3′, antisense 5′-UUGGAACCUGGCUAUAUGGTT-3′. All primers were synthesized by GenePharma. LMH cells were transfected using Lipofectamine RNAiMAX (Invitrogen) according to the manufacturer’s protocol. The efficiency of RNA silencing was verified 48 h after transfection by qPCR. To overexpress chicken CAR, LMH cells were transfected with the pCAGGS-CAR using jetPRIME (Polyplus-transfection) following the manufacturer’s protocol.

### Statistical analysis

The data shown are expressed as the mean ± standard deviation (SD) and were analyzed by analysis of variance, as implemented in SPSS software (version 19.0). Differences were considered statistically significant at *p < 0.05 and **p < 0.01.

## Results

### Fibre 1 was identified as the short fibre of the novel FAdV-4

**Figure 1.**
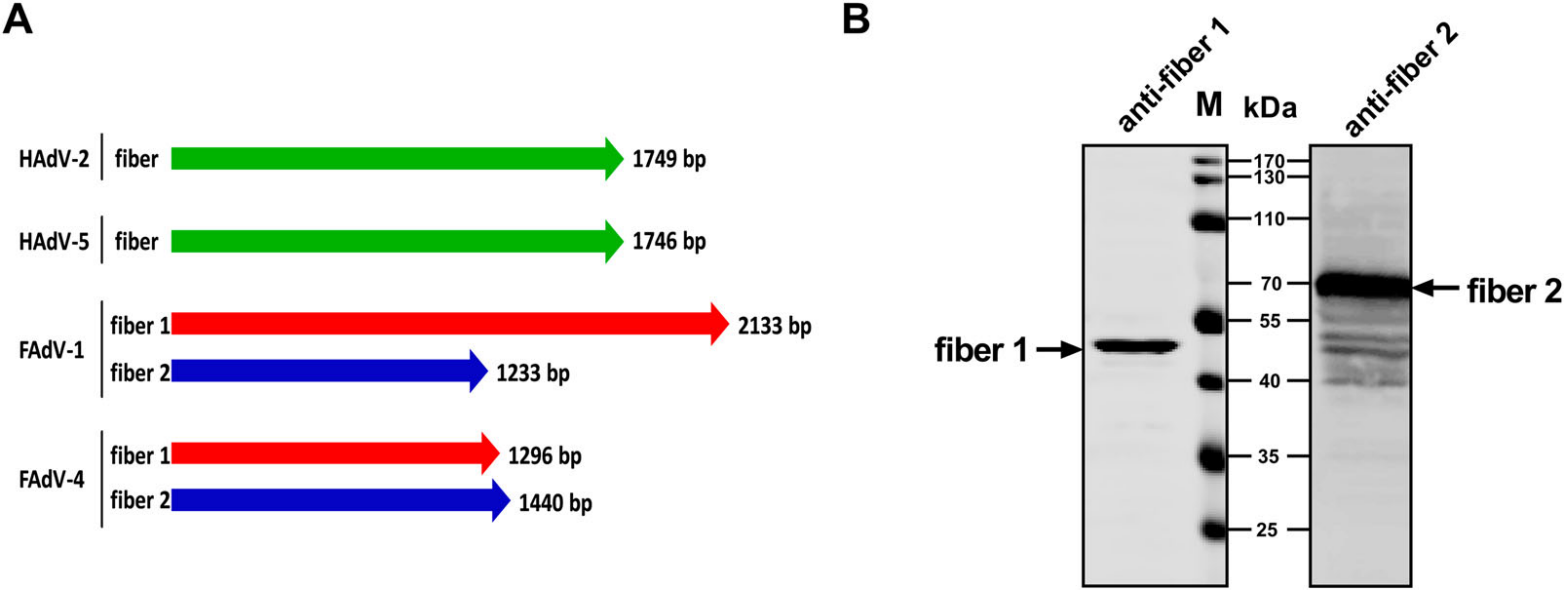
Fibre 1 is the short fibre protein of the novel FAdV-4. (A) The mode pattern of distinct fibres in different AdVs. (B) LMH cells were infected with FAdV-4 at a MOI of 0.1 and a cell lysate was prepared for western blot analysis, using pAbs against fibre 1 and fibre 2. The fibre 2 protein (70 kDa) was clearly larger than fibre 1 (45 kDa).

The complete genome sequences encoding various AdVs fibres were aligned (Figure 1(A)). Most human AdVs (such as human HAdV-2 and HAdV-5) have one unique fibre, whereas FAdV-1 and FAdV-4 have two distinct fibres inserted into each penton. Nevertheless, the fibre patterns of FAdV-1 and FAdV-4 were variable. CELO (FAdV-1) was found to have long fibre 1 protein of 710 aa and a short fibre 2 protein of 410 aa. In contrast, the novel FAdV-4 fibre 1 and 2 proteins were found to contain 431 and 479 aa, respectively. Therefore, the fibre 1 protein of the novel FAdV-4 was predicted to be the short fibre, based on the aa sequences. To confirm the native protein sizes of fibres 1 and 2 of FAdV-4, leghorn male hepatocellular (LMH) cells were inoculated with FAdV-4 at a multiplicity of infection (MOI) of 0.1 for 48 h, after which a lysate was prepared for western blot analysis, using polyclonal antibodies (pAbs) against fibre 1 or fibre 2. The results (Figure 1(B)) showed bands for fibre 1 (∼45 kDa) and fibre 2 (∼70 kDa), indicating that the fibre 1 protein functioned as the short fibre protein of FAdV-4 in natural virions.

### The short fibre 1 protein determined the infectivity of FAdV-4

**Figure 2.**
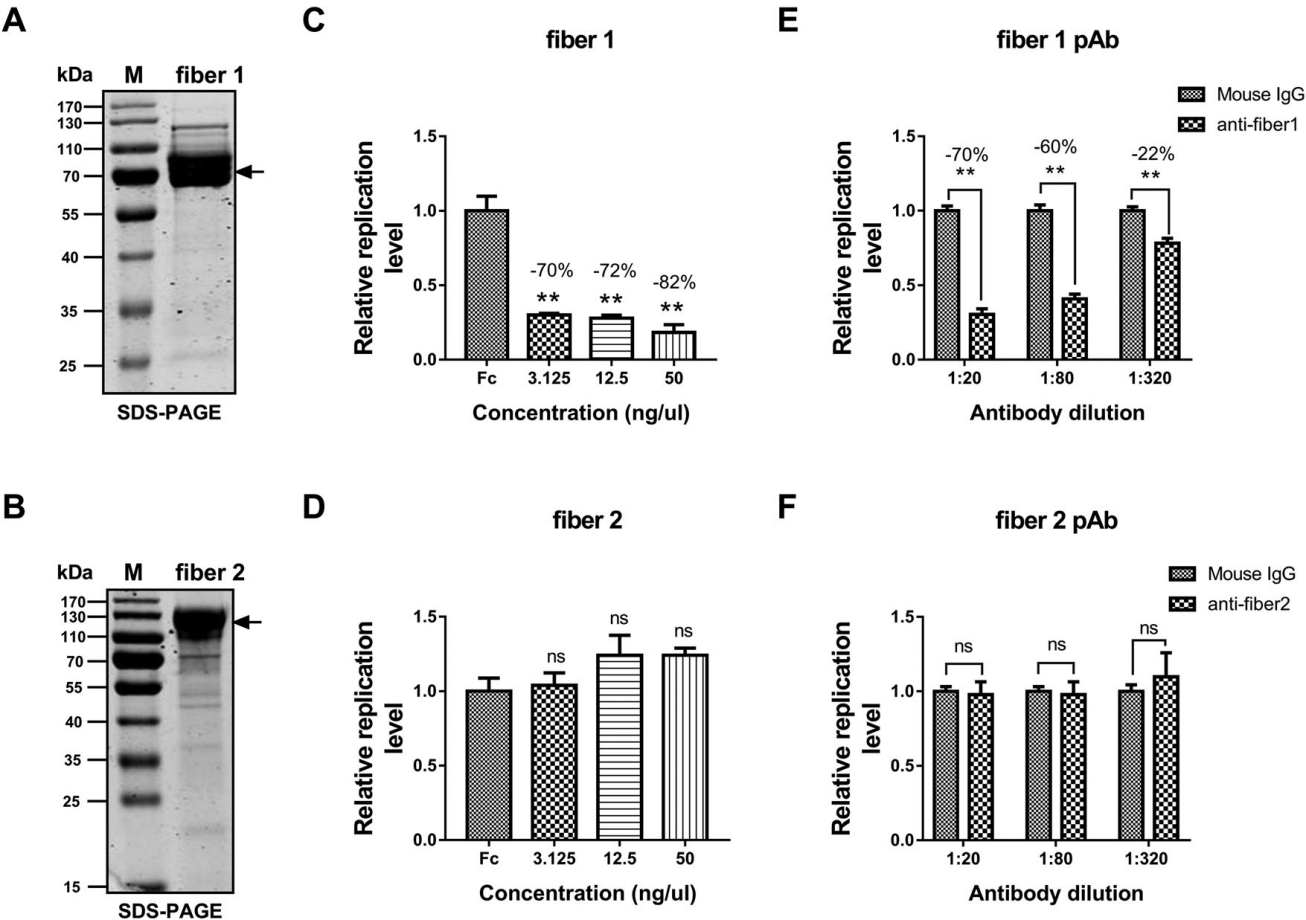
Fibre 1 determined the infectivity of FAdV-4. SDS-PAGE analysis of purified soluble fibre 1 (A) and fibre 2 (B) proteins. Different concentrations (3.125, 12.5, or 50 ng/μl) of the fibre 1 or fibre 2 protein were pre-incubated with LMH cells at 37°C for 1 h. Next, the cells were inoculated with FAdV-4 (MOI = 0.1) and further incubated for 1 h at 37°C. After washing the cells three times with PBS, they were cultured for 24 h under normal culture conditions. Finally, the samples were collected for qPCR analysis. The fibre 1 protein significantly blocked the infectivity of FAdV-4 (*P* < 0.05) (C), but the fibre 2 protein did not (*P* ≥ 0.05) (D). Different dilutions of antibodies against fibre 1 or fibre 2 (or mouse IgG as a negative control) were incubated with 0.1 MOI FAdV-4 at 37°C for 1 h and then washed twice with PBS. Thereafter, the mixture was added to LMH cells and incubated for another 1 h at 37°C. After washing the cells three times, they were maintained under normal culture conditions and collected at 24 hpi for qPCR analysis. The antibody against fiber1 (E), but not fibre 2 (F), blocked FAdV-4 infection in a concentration-dependent manner. The data are presented as the mean ± SD of at least three independent experiments.

To identify the fibre that determines FAdV-4 infectivity, antibody-blockade assays were performed with purified recombinant proteins. The soluble fibre 1 (Figure 2(A)) and fibre 2 (Figure 2(B)) proteins were expressed and purified by affinity chromatography. The purified soluble fibre 1 and 2 proteins were then studied in competitive-inhibition experiments. LMH cells were pre-incubated with different concentrations (3.125, 12.5, and 50 ng/μl) of purified fibre 1 and fibre 2 proteins, and then the cells were inoculated with FAdV-4 (MOI = 0.1) for 24 h. Thereafter, the samples were collected for quantitative PCR (qPCR) analysis. The negative control was normalized to 1, and the relative infection level was calculated as described. The results showed that the different concentrations of fibre 1 protein tested blocked the infectivity of the novel FAdV-4 from 70% to 82% (Figure 2(C); *P* < 0.01). In contrast, the fibre 2 protein did not block infectivity at any concentration tested (Figure 2(D); *P* ≥ 0.05).

### An anti-fibre 1 antibody neutralized FAdV-4 infection

In another experiment, different concentrations of anti-fibre 1 and anti-fibre 2 pAbs were used in the blockade assays. Briefly, different dilutions of antibodies were pre-incubated with FAdV-4 and then added to LMH cells for an MOI of 0.1. The cell samples were harvested at 24 h post-infection (hpi) for qPCR analysis, as described above. Consistent with the results mentioned above, the anti-fibre 1 antibody significantly neutralized infection by the novel FAdV-4 in a concentration-dependent manner, ranging from 22% to 70% inhibition (Figure 2(E); *P* < 0.01). However, the anti-fibre 2 antibody did not neutralize FAdV-4 infection (Figure 2(F); *P* ≥ 0.05). Taken together, these findings indicate that FAdV-4 infection was determined by fibre 1 instead of fibre 2.

### Testing CAR as a protein that interacts with the FAdV-4 fibre 1 protein

**Figure 3.**
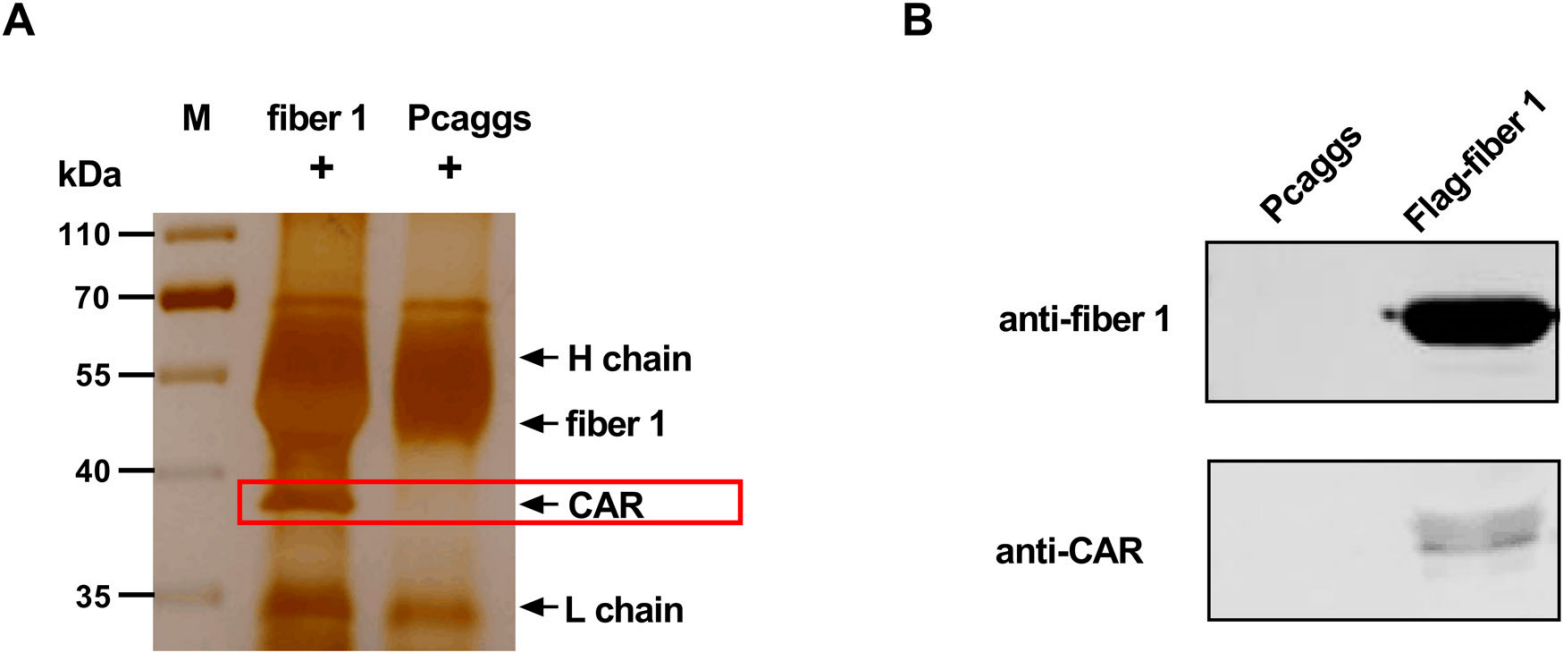
The chicken CAR homolog was identified as a protein that interacts with FAdV-4 fibre-1. Fibre 1 interaction with cellular proteins were analyzed by LC/MS and confirmed by western blotting. (A) LMH cell lysates were incubated with Fc-tagged fibre 1 or Fc protein (direct bound to protein A/G) for 6 h and then subjected to SDS-PAGE. An additional band was observed by silver staining in the fibre 1 sample, compared to the negative control sample. The band was identified as the chicken CAR homolog (red box) by LC/MS. (B) The band was further confirmed to be CAR by performing western blot analysis with a CAR-specific antibody.

Although the fibre 1 protein of the novel FAdV-4 was critical for infection, the cellular receptor of the novel FAdV-4 was unknown. To screen potential receptor candidates, mock LMH cell lysates were mixed with protein A/G sepharose beads that were preincubated with an Fc-tagged anti-fibre 1 antibody. The proteins immunoprecipitated with the anti-fibre 1 antibody were analyzed by LC/MS, which enabled detection of chicken CAR as a ∼38-kilodalton (kD) band visualized by silver staining (Figure 3(A)). Confirmation that chicken CAR was immunoprecipitated with the anti-fibre 1 antibody was determined by western blot analysis with a chicken CAR-specific antibody (Figure 3(B)).

### Functional identification of chicken CAR as a cellular receptor for the novel FAdV-4

**Figure 4.**
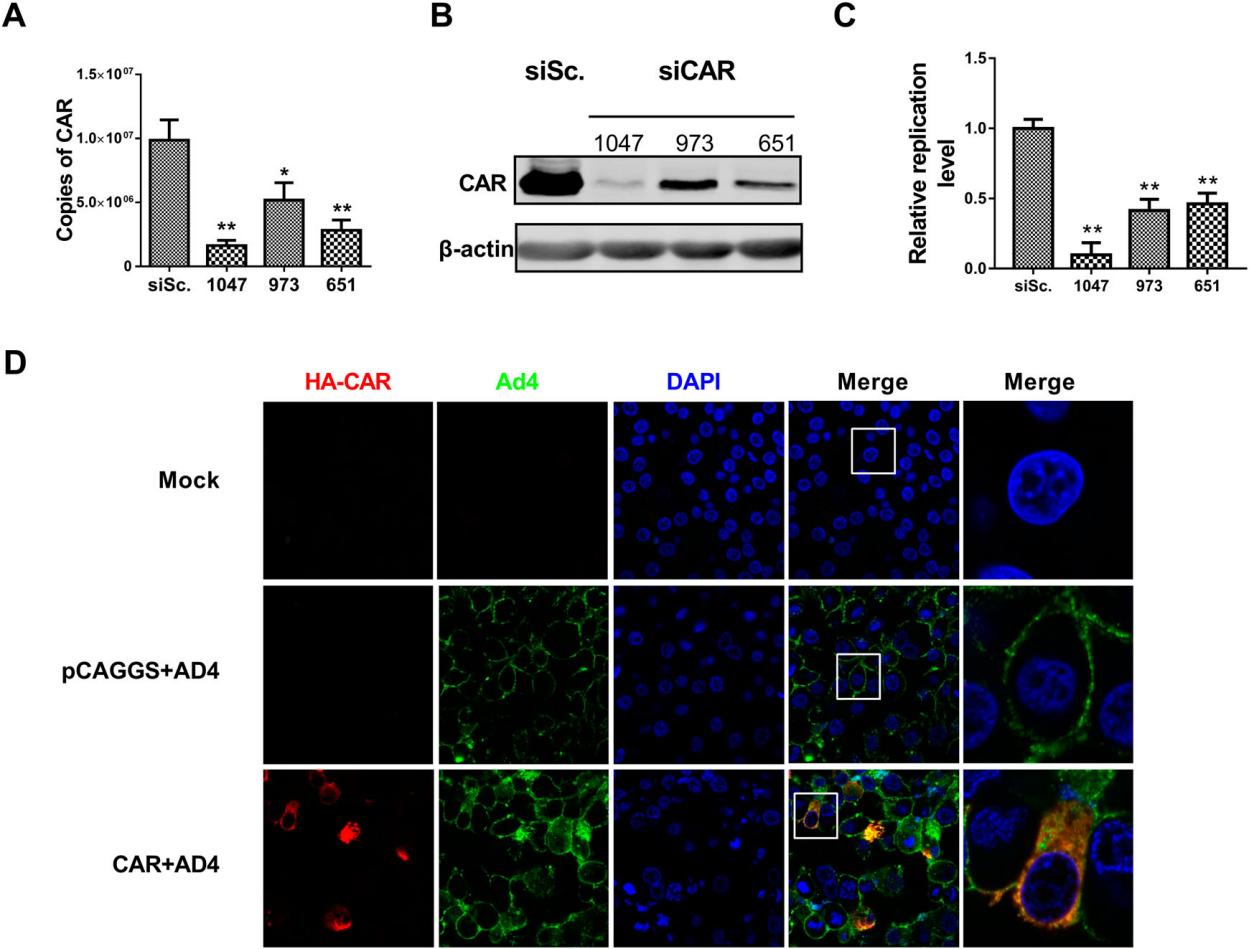
CAR facilitated FAdV-4 infection. To determine whether CAR influenced the infectivity of FAdV-4, RNAi and overexpression assays was performed. (A) All of the CAR siRNAs tested effectively downregulate the mRNA transcription of CAR. (B) The tested siRNAs effectively depleted CAR protein expression. (C) After pre-treatment with CAR siRNA for 24 h, LMH cells were inoculated with FAdV-4 (MOI = 0.1) and the relative infection levels were measured after 24 h. FAdV-4 infection was significantly inhibited by RNAi (*p* < 0.05). (D) CAR overexpression promoted the infectivity of FAdV-4. FAdV-4 (MOI = 1) was incubated with CAR-overexpressing LMH cells for 1 h and then the distribution of the virus was analyzed. FAdV-4 (green fluorescence) was located on the membrane of mock-treated LMH cells. However, following CAR overexpression (red fluorescence), FAdV-4 migrated into the cytoplasm and co-localized with the CAR protein. The data shown are presented as the mean ± SD of at least three independent experiments.

To further study whether CAR influences the infectivity of FAdV-4, RNA-interference (RNAi) assays were performed with small-interfering RNAs (siRNAs). All siRNAs tested effectively downregulate CAR mRNA (Figure 4(A)) and protein (Figure 4(B)) expression. After 24 h of RNAi, the LMH cells were infected with FAdV-4 (0.1 MOI) and the relative infection levels were tested, as described above. CAR RNAi significantly inhibited FAdV-4 infection (Figure 4(C); *P* < 0.05). Moreover, CAR overexpression was tested to confirm whether it could promote FAdV-4 infectivity. LMH cells were transfected with pCAGGS-CAR or the empty vector (negative control) for 24 h and subsequently incubated for 1 h with FAdV-4 (MOI = 1) at 4°C. Virus internalization was observed by laser-scanning confocal microscopy after staining with a rabbit anti HA tag antibody to detect HA-tagged CAR and an anti-Hexon monoclonal antibody (Mab) against FAdV-4. The results showed that FAdV-4 (green fluorescence) localized to the membrane of LMH cells in empty vector-transfected control samples, but migrated into the cytoplasm and co-localized with the CAR protein (red fluorescence) in CAR-overexpressing cells (Figure 4(D)). Collectively, these results showed that the infectivity of the novel FAdV-4 correlated with the expression level of the chicken CAR protein.

### Both soluble CAR and a CAR-specific antibody blocked FAdV-4 infection

**Figure 5.**
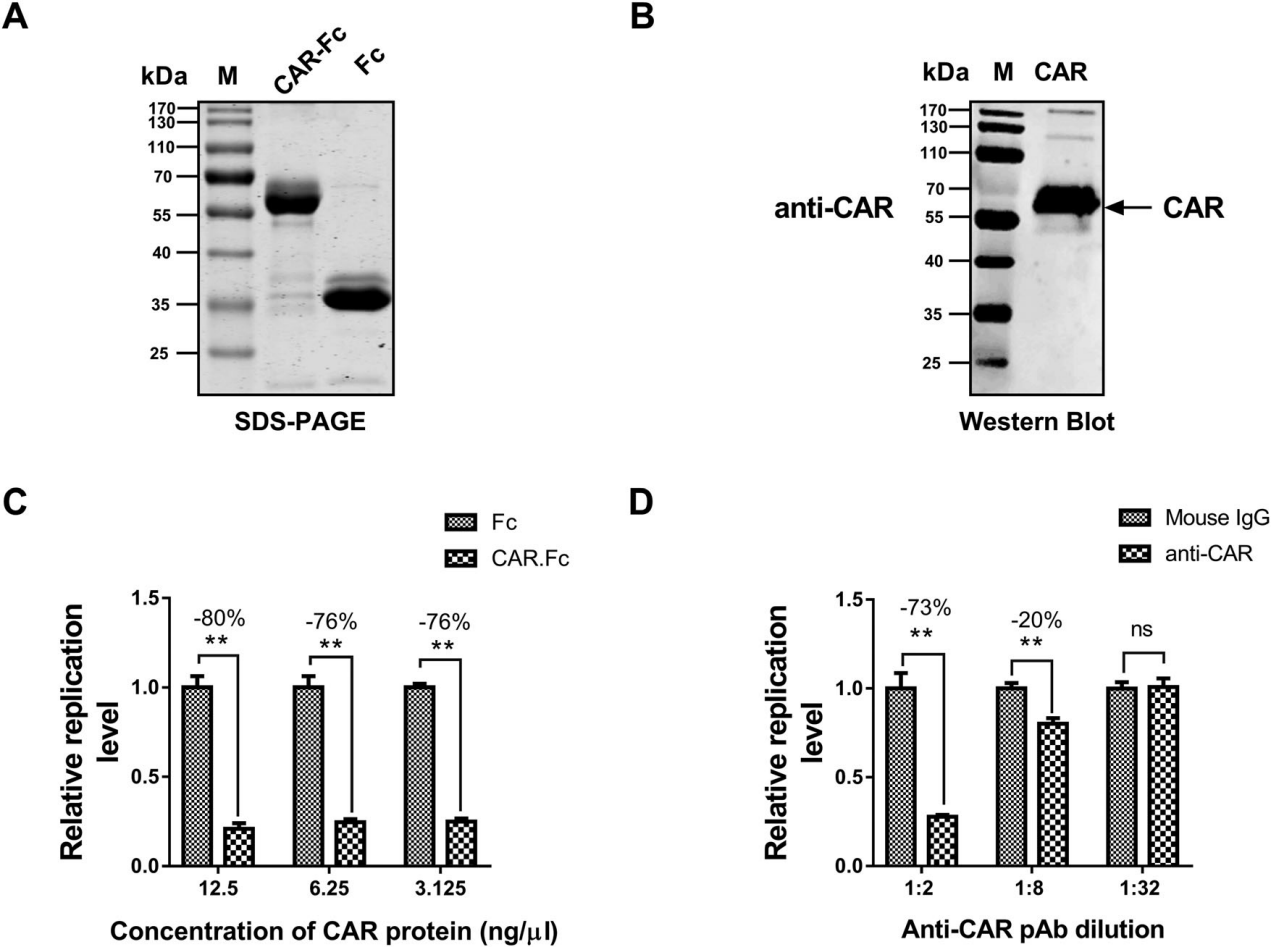
Both soluble CAR and a CAR-specific antibody blocked the infection of FAdV-4. The proper expression and purification of Fc-tagged CAR were identified by SDS-PAGE (A) and western blotting (B). (C) Different concentrations of the soluble CAR protein, ranging from 3.125 to 12.5 ng/μl (with the Fc protein serving as a negative control), were used in the protein-based blockade assay. All detected concentrations of soluble CAR protein could block FAdV-4 infection by 76% to 80% (*p* < 0.05). (D) In the antibody-based blockade assay, we applied different dilutions of the CAR pAb, ranging from 1:2 to 1:32, with mouse IgG serving as a negative control. The CAR antibody could block the infectivity of FAdV-4 in a concentration-dependent manner (1:2 dilution, 73% inhibition; 1:8 dilution, 20% inhibition; 1:32 dilution, 0% inhibition) (*p* < 0.05). The data shown are presented as the mean ± SD of at least three independent experiments.

To further confirm the receptor function of chicken CAR for the novel FAdV-4, blockade assays were conducted using a soluble CAR protein or an anti-CAR antibody. Eukaryotic expression and purification of the Fc-tagged CAR protein was verified by sodium dodecyl sulfate-polyacrylamide gel electrophoresis (SDS-PAGE; Figure 5(A)) and western blotting (Figure 5(B)). In the protein-blockade assays, different concentrations of soluble CAR protein (3.125–12.5 ng/μl; Fc protein as the negative control) were used. The experiments were performed in a manner similar to that described above for blocking with the fibre antibody. The results indicated that all detected concentrations of the soluble CAR protein blocked FAdV-4 infection by 76–80% (*P* < 0.05; Figure 5(C)). In the CAR antibody-blockade assay, we applied different dilutions of the CAR pAb (ranging from 1:2 to 1:32; mouse IgG as a negative control). The experiment was performed with blocking using a fibre protein antibody, as described above. As shown in Figure 5(D), the CAR antibody significantly (*P* < 0.05) blocked FAdV-4 infectivity in a concentration-dependent manner (1:2 dilution, 73% blocking; 1:8 dilution, 20% blocking; 1:32 dilution, 0% blocking.

### CAR expression enabled the novel FAdV-4 to enter non-permissive cells

**Figure 6.**
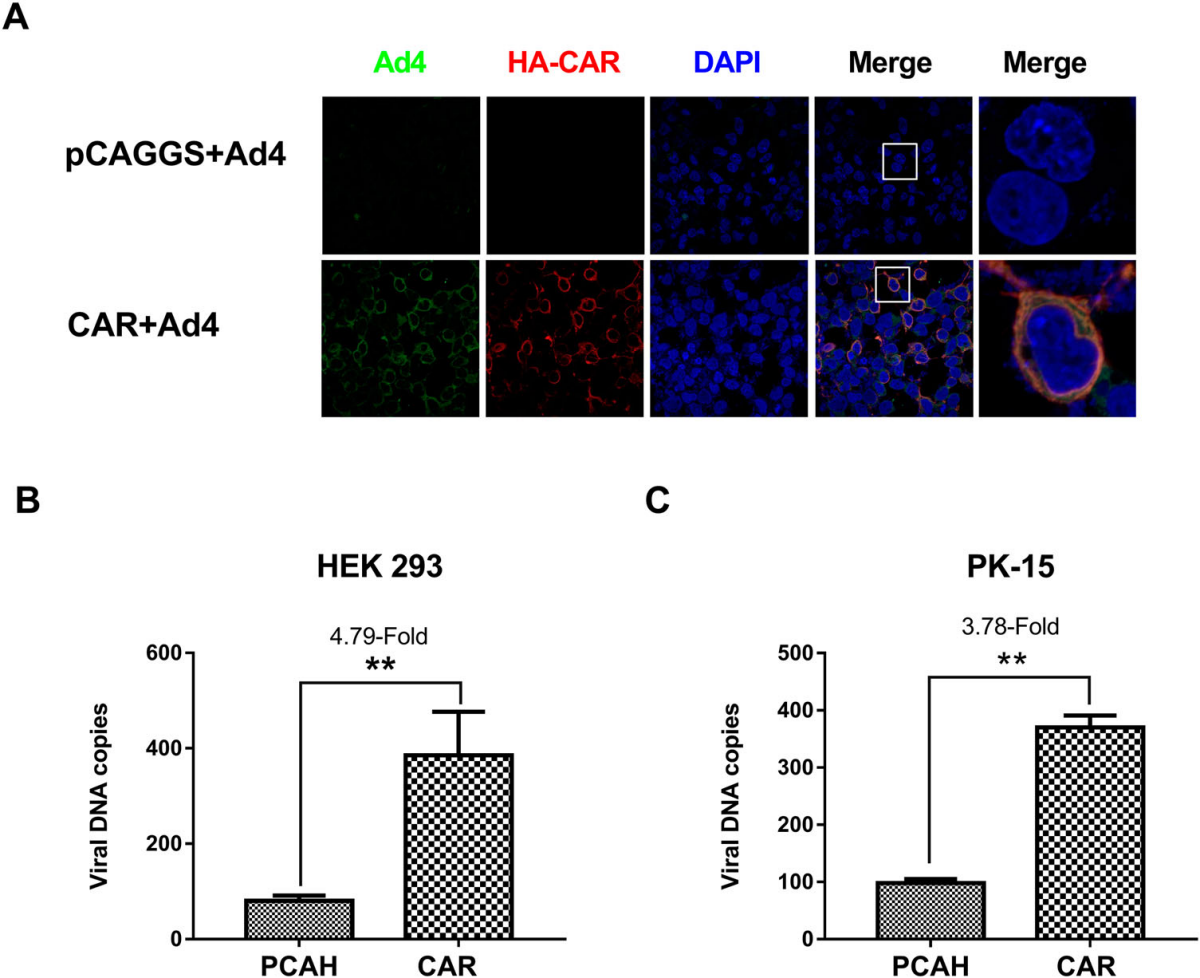
CAR conferred FAdV-4 entry into non-permissive cells. (A) Non-permissive 293 T cells were transfected for 24 h with a pCAGGS vector driving HA-tagged CAR overexpression (empty pCAGGS vector, negative control), followed by FAdV-4 infection (MOI = 1) for 24 h. The samples were analyzed by indirect immunofluorescence using rabbit antibodies against the HA tag and the FAdV-4 Hexon protein. FAdV-4 (green fluorescence) co-localized with CAR (red fluorescence) in the cytoplasm of 293 T cells, whereas virus and CAR were not detected in the negative-control cells. CAR overexpressing 293 T cells (B) and PK-15 cells (C) were infected with FAdV-4 (MOI = 1) and collected for qPCR detection at 24 hpi. The results showed that non-permissive cells could confer viral entry into cells, with the viral load increased by 4.79-fold in 293 T cells and 3.78-fold in PK-15 cells (*P* < 0.05). The data shown are presented as the mean ± SD of at least three independent experiments.

To further assess whether CAR could confer susceptibility to FAdV-4 infection in non-permissive cells, 293 T cells were transfected for 24 h with a pCAGGS vector encoding HA-tagged CAR or the pCAGGS empty vector (negative control). Thereafter, the cells were infected with FAdV-4 (MOI = 1) for another 24 h. The samples were fixed and the virus distribution was observed using a laser-scanning confocal microscopy after staining with a rabbit anti-HA tag antibody for CAR and an anti-FAdV-4 Hexon Mab. The results showed that FAdV-4 (green fluorescence) co-localized with HA-tagged CAR (red fluorescence) in the plasma of 293 T cells. No virus was detected in uninfected control cells, although HA-tagged CAR was detected (Figure 6(A)). In another experiment, non-permissive 293 T and PK-15 cells were transfected with a pCAGGS vector driving HA-tagged CAR overexpression or the empty pCAGGS vector for 24 h before infection with FAdV-4 (MOI = 1). Samples were collected for qPCR detection at 24 hpi. Our results showed that CAR overexpression in non-permissive cells enabled viral entry, with a 4.79-fold increase in viral load in 293 T cells (Figure 6(B)) and a 3.78-fold increase in viral load in PK-15 cells (Figure 6(C)) (*P* < 0.01).

### The D2 domain of CAR was identified as the active domain for binding the novel FAdV-4

**Figure 7.**
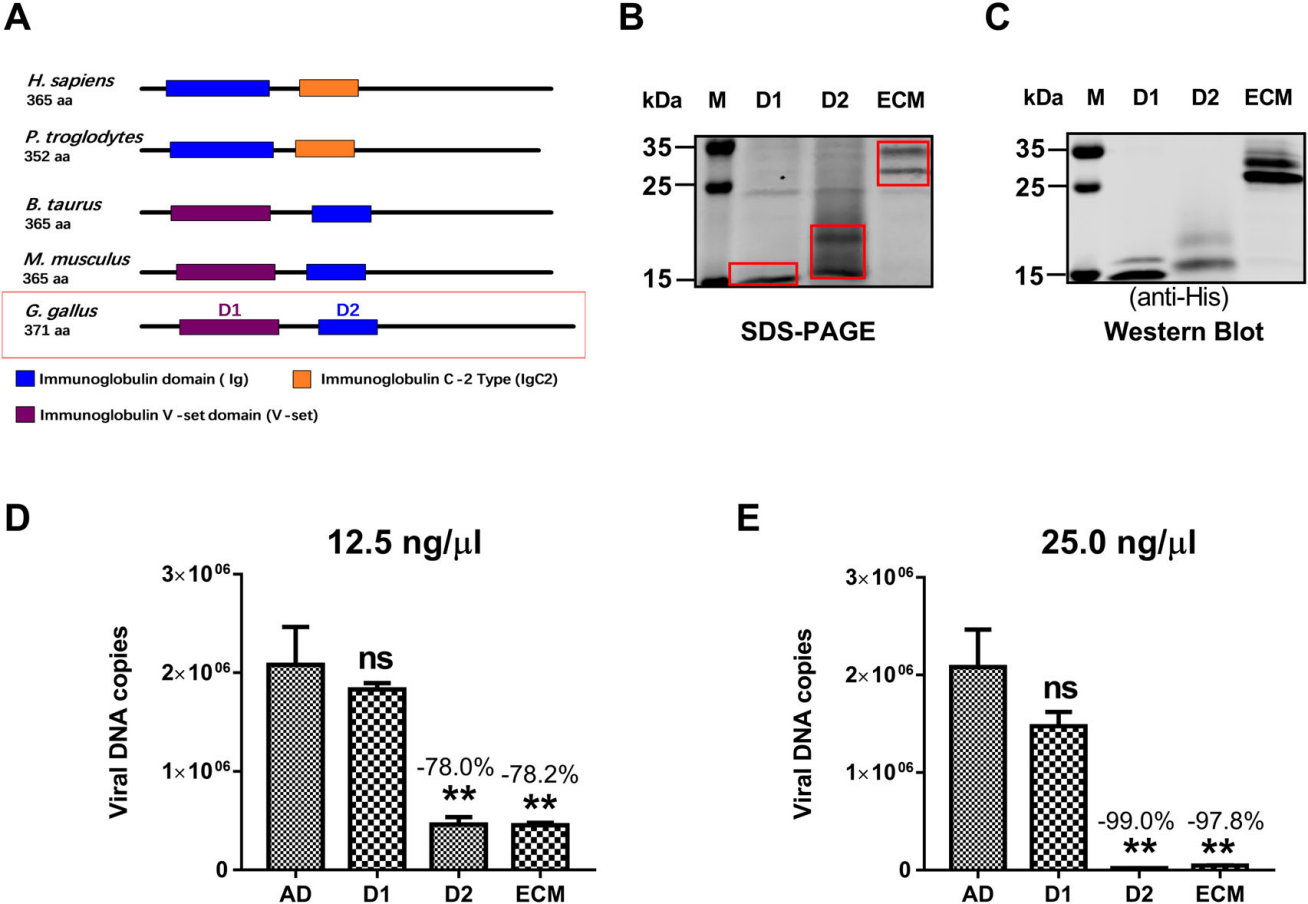
The D2 domain of CAR enabled FAdV-4 infection. (A) The mode pattern of CAR functional domains in different species. The *Gallus* CAR Ig V-set domain (D1) and the Ig domain (D2) are shown in the red box. (B) SDS-PAGE detection of soluble His-tagged variants of the D1, D2, and ECD (D1 + D2) domain (red box). (C) Western blot-based identification of the D1, D2, and ECD domains of CAR. Soluble CAR D1, D2, and ECD proteins were used in the protein-based blockade assay. CAR D2 at concentrations of 12.5 ng/μl (D) and 25 ng/μl (E) block the infectivity with efficacies of 78% and 99.9% (*P *< 0.05), respectively, in contrast to the D1 domain (which did not block infection). The data shown are presented as the mean ± SD of at least three independent experiments.

To further investigate which functional domain of CAR affected FAdV-4 infectivity, we expressed and purified soluble, His-tagged proteins, corresponding to the CAR immunoglobulin V-set domain (D1 domain), the immunoglobulin domain (D2 domain), and the extracellular domain (ECD) (Figure 7(A and B), red boxes) from eukaryotic cells, as described above for the fibre 2 protein and confirmed by western blotting using an antibody against the His-tag (Figure 7(C)). To determine which of the functional domains were responsible for FAdV-4 infectivity we used, 12.5 ng/μl (Figure 7(D)) or 25.0 ng/μl (Figure 7(E)) of His-tagged ECD, D1-CAR, and D2-CAR proteins in blockade assays, which were performed similarly to that described above for the CAR protein. The observations indicated that both 12.5 and 25.0 ng/μl of D2-CAR could block the infectivity of FAdV-4 from 78% to 99.9% (*P* < 0.01), in contrast to D1-CAR and the negative control. These findings demonstrated that the D2-CAR acted as the active domain and affected the infectivity of the novel FAdV-4.

## Discussion

The initial recognition and binding of AdVs to the host cell surface is mediated by the fibre protein. Usually, AdVs use the unique fibre [13] or the long fibre [29,30] to bind the cell surface receptor, although the fibre patterns of FAdVs clearly differ from those of other AdVs, and the fibre patterns of the twelve FAdV serotypes also showed high diversity. Notably, FAdV-1 and FAdV-4 have two distinct fibres inserted into each penton base, which is clearly different from AdVs of other species. The complete genome sequences showed that the fibre 1 protein of FAdV-1 (710 aa) is clearly longer than the fibre 2 protein (410 aa), whereas the lengths of the fibre proteins of the novel FAdV-4 were relatively equal (431 aa for fibre 1 and 479 aa for fibre 2). In this study, fibre 1 of the novel FAdV-4 was identified as a shorter fibre than fibre 2, based on the number of aa, as well as the protein size in the natural virus. Moreover, some modifications of fibre 2 may have occurred, which further increased the molecular weight more than predicted. The crystal structure of the fibres and the potential modifications of fibre 2 need to further investigated, as these modifications were important for identifying the unique fibre pattern of the novel FAdV-4.

Given the clearly different fibre patterns of the emerging novel genotype FAdV-4 from other AdVs and CELO (an FAdV-1 vector) [31,32], a novel fibre–receptor-interaction mechanism might promote FAdV-4 infection. To test this hypothesis, we purified both fibres proteins of the novel FAdV-4, and the short fibre was found to play critical roles in binding to susceptible LMH cells in protein-blockage and antibody-neutralization assays. In contrast, the other Ads utilized the unique fibre and CELO used the long fibre to infect host cells. The long fibre 2, coupled with the Hexon protein, has been closely associated with the virulence of the emerging FAdV-4 [33]. To our knowledge, this is the first report describing the cellular-binding function of the short fibre of FAdV-4. Our findings also revealed a novel AdV-receptor-binding mechanism and highlighted potential new targets for developing new vaccines and antiviral drugs for the control of the emerging highly pathogenic FAdV-4.

Variable cellular receptors have been identified for adenovirus [13,16,21], but the cellular receptor of the novel FAdV-4 has not been identified. In this study, the purified short fibre was used to screen for interacting cell-surface proteins. Subsequently, the chicken CAR homolog was immunoprecipitated with the purified short fibre and the CAR protein was identified as a cellular receptor for FAdV-4 by CAR overexpression, RNAi, and blockade experiments with purified CAR or an anti-CAR antibody. Although human CAR was characterized as a cellular receptor for subgroup C [13] and subgroup F [29] adenoviruses, and group B coxsackieviruses [34,35], the chicken CAR homolog was identified here for the first time as a cellular receptor for the novel FAdV-4. The receptor function was further confirmed by overexpressing CAR in non-permissive HEK 293 and PK-15 cells, which efficiently facilitated the infectivity of FAdV-4. Although FAdV-1 also uses CAR as a cellular receptor (26), the binding mechanism is clearly different. FAdV-1 uses its long fibre to bind CAR on the cell surface, whereas FAdV-4 utilized the relatively shorter fibre. Notably, blockage assays performed with purified CAR protein or a pAb against CAR showed high inhibition (80% and 73% respectively), indicating that chicken CAR plays a critical role in FAdV-4 infection, although FAdV-4 may use other receptors and further studies are needed to explore this possibility.

As reported, CAR is a transmembrane protein containing an extracellular domain composed of two immunoglobulin-like domains, a single transmembrane domain, and an intracellular domain [36,37]. The distal Ig-like D1 domain mediates hemophilic interactions and is responsible for high-affinity binding with the AdV fibre protein, whereas the proximal Ig-like D2 domain is required for AdV fibre-knob binding and infection [36,38]. In this study, the proximal D2 domain of CAR was identified as the active binding domain, based on data showing that direct binding of FAdV-4 to purified D2-CAR significantly decreased viral infectivity in susceptible LMH cells. Although different CAR homologs showed 56.8–64.0% aa identity, different CAR proteins had similar structures and location in host cells [39,40], which might explain its utility by various types of AdVs in infecting different hosts. Nevertheless, the distal D1 domain of CAR is usually reported to directly mediate homophilic interactions responsible for high-affinity binding to AdV fibre proteins [36,38,41]. However, our findings revealed a novel interaction pattern between the short fibre of the novel FAdV-4 and the proximal D2 domain of chicken CAR, which is important for further understanding the molecular details between AdV fibre proteins and CAR.

In conclusion, fibre 1 was characterized as a short fibre protein of the emerging novel FAdV-4 that was critical for binding the cellular receptor CAR, which was important for FAdV-4 infection. This finding differs from those of previous studies showing that AdV utilized the unique or long fibre to bind surface molecules on host cells. Furthermore, the chicken CAR protein was identified as a cellular receptor of the emerging FAdV-4 for the first time by detecting interactions between the proximal D2 domain and the short viral fibre 1 protein. Finally, a novel binding mechanism of AdV infection was revealed for the novel FAdV-4, which provides important information for developing new vaccines and antiviral drugs for the emerging HHS.
